# “For a Long Time Our Voices have been Hushed”: Using Student Perspectives to Develop Supports for Neurodiverse College Students

**DOI:** 10.3389/fpsyg.2017.00544

**Published:** 2017-04-18

**Authors:** Kristen Gillespie-Lynch, Dennis Bublitz, Annemarie Donachie, Vincent Wong, Patricia J. Brooks, Joanne D’Onofrio

**Affiliations:** ^1^Department of Psychology, College of Staten Island and The Graduate Center, The City University of New York, New YorkNY, USA; ^2^Department of Psychology, College of Staten Island, The City University of New York, New YorkNY, USA; ^3^Center for Student Accessibility, College of Staten Island, The City University of New York, New YorkNY, USA

**Keywords:** disabilities, autism, college students, self-advocacy, social skills, universal design

## Abstract

Although the challenges that autistic students face adapting to college are often pronounced, they are similar to the challenges that students with other disabilities face (e.g., difficulties with social interaction, self-advocacy, and executive functioning). However, extant evaluations of services for autistic college students are very limited despite an emerging literature examining supports for college students with a range of other disabilities. Given that many autistic students do not self-identify as autistic in college, and consequently might avoid autism-specific services, autistic students might benefit from services that are designed to support a broad range of neurodiverse students, or services that are structured according to the principles of Universal Design. In order to develop such services, we assessed the self-reported needs of autistic college students and their peers with other disabilities. Guided by needs assessments and feedback from students, we developed and evaluated two semesters of mentor-led group programming for autistic college students and students with other disabilities. The first semester of the program focused on social skills; after receiving feedback from participants, the curriculum for the second semester focused on self-advocacy. Participation in social-skills groups was associated with decreased anxiety and autism symptoms. Participation in self-advocacy groups was associated with increased perceived social support from friends, academic self-efficacy, and more accurate definitions of self-advocacy. This research suggests that supports for neurodiverse college students should be developed with their input and should include opportunities to engage with diverse peers.

## Introduction

Although the symptoms of Autism Spectrum Disorder (ASD) tend to improve from adolescence into adulthood (Shattuck et al., 2007), autistic^1^ individuals often struggle with transitioning into college and the workforce (Van Bergeijk et al., 2008; Hendricks, 2010; Kapp et al., 2011). Many autistic adults have few or no close social relationships outside of the family, are unable to live independently, and are either unemployed or employed in jobs that are not commensurate with their skills (Howlin et al., 2004; Shattuck et al., 2011a; Hillier and Galizzi, 2014). These poor outcomes stand in stark contrast to the viewpoint shared by many autistic self-advocates, parents, and professionals that autistic adults have the potential to contribute substantially to society (e.g., Prince, 2010; Autistic Self-Advocacy Network, 2011; Wehman et al., 2014).

One potential contributor to poor outcomes among autistic adults is a lack of supports; services available to autistic children and adolescents are often no longer available to adults (Shattuck et al., 2011b). Consequently, autistic adults who are intellectually capable, and often gifted, may spend their time in solitary unstructured activities (e.g., watching TV). College education can provide opportunities for autistic adults to be members of a community while developing the skills needed to gain independence. Autism is associated with a number of strengths that can help autistic students succeed in college, including high intrinsic motivation, attention to detail, memory skills, systematic thinking, ability to develop productive routines, intense interests, and sincerity (Gobbo and Shmulsky, 2014; Schindler et al., 2015; Van Hees et al., 2015).

Despite these strengths, autistic students often struggle with transitioning from high school to the less structured and more socially complex college environment (e.g., Kapp et al., 2011). High school graduates with autism may fail to enter college or drop out before completing their degrees (Glennon, 2001; Cederlund et al., 2008; Van Bergeijk et al., 2008). Indeed, young adults with an educational classification of autism are less likely to enroll in 2- and 4-year colleges than students in all other disability categories except intellectual disabilities or multiple disabilities (Wei et al., 2013). While 70% of recent high school graduates were enrolled in college in 2009, only 32% of recent high school graduates with an educational classification of autism were enrolled in college (US Census Bureau, 2012; Wei et al., 2013). Low college enrollment is particularly apparent among autistic students from economically disadvantaged families.

Although autistic students are less likely to enroll in college than their peers with other disabilities, anecdotal evidence suggests that the number of autistic college students is increasing (e.g., Van Bergeijk et al., 2008). However, limited research has focused on this population. In the first (and currently only) peer-reviewed study to examine the prevalence of ASD among college students, White et al. (2011) found that 0.7% of the students at a public university met diagnostic criteria for ASD, yet none had previously been diagnosed with ASD. Determining the prevalence of ASD among college students is complicated by voluntary disclosure of diagnosis: Among college students who were identified as autistic in high school, approximately 37% chose not to identify themselves as autistic to their colleges (Newman et al., 2011), which suggests that they may not reach out for help until problems arise (MacLeod and Green, 2009).

Autistic individuals may experience significant challenges navigating college life, which greatly impact their ability to function effectively on campus and in the workplace post-graduation (Adreon and Durocher, 2007; Cimera and Cowan, 2009). These difficulties include atypical sensory processing, inflexibility, executive function difficulties, challenges engaging in self-advocacy, social difficulties, depression, and anxiety (Glennon, 2001; Van Bergeijk et al., 2008; White et al., 2011; Schindler et al., 2015; Cai and Richdale, 2016). The stress that most students experience when transitioning into college may be compounded for autistic students, as difficulty in coping with change is part of the diagnostic criteria for ASD. Loss of previously provided supports may also provoke anxiety (Glennon, 2001).

In response to evidence that autistic students may need specialized supports to succeed in college, an emerging body of literature provides recommendations for how to support college students on the spectrum (Glennon, 2001; Adreon and Durocher, 2007; Van Bergeijk et al., 2008; MacLeod and Green, 2009; Wenzel and Rowley, 2010; Kapp et al., 2011; Pillay and Bhat, 2012; Gobbo and Shmulsky, 2014; Zeedyk et al., 2014; Burgstahler and Russo-Gleicher, 2015; Van Hees et al., 2015). These recommendations have typically been based on literature reviews, case studies, and the insights of those who work with students on the spectrum. They stress the importance of individualized supports, such as mentoring, to help students on the spectrum develop self-advocacy, social, and executive functioning/self-regulation skills. However, only a few studies have directly assessed the needs and experiences of more than a few college students on the spectrum (Gelbar et al., 2014; Van Hees et al., 2015; Cai and Richdale, 2016; Roberts and Birmingham, 2017).

Consistent with the paucity of research about college students on the spectrum, services for autistic college students remain very limited (Shattuck et al., 2012a,b; Barnhill, 2016). Currently, there are no intervention programs with an established evidence base to facilitate transitions into college, and from college into the workplace, for individuals on the autism spectrum (Bishop-Fitzpatrick et al., 2013). A recent Internet search and review of the literature identified 31 postsecondary institutions in the United States that confirmed that they had specialized autism support services (Barnhill, 2016). Although most of the programs charged a fee for services (fees averaged $6525 a year), few of the programs collected outcome data. Therefore, it is essential to develop and evaluate supports for autistic college students.

Although evidence-based supports for autistic college students are greatly needed, extant peer-reviewed research (as of February, 2017) contains preliminary evaluations of only *three* college-based programs to support autistic college students. Since 2005, autistic students at a liberal arts college have received one-on-one mentorship from occupational therapy graduate students (Schindler et al., 2015). Standardized interviews conducted with 11 autistic students at the beginning and end of two terms of the program revealed self-reported improvements in executive functioning and socialization. In response to an influx of students on the autism spectrum, a clinical psychologist founded a mentorship program for autistic college students at York University in 2007 (described in more detail in the program manual; Bebko et al., 2011). Each autistic student in the program is paired with a one-on-one graduate student mentor. Students are also invited to attend group social activities (e.g., “pizza parties” or university sports events) and workshops (e.g., about sexuality or managing exam stress). Preliminary findings from this program were recently published (Ames et al., 2016; Roberts and Birmingham, 2017). Twelve autistic mentees completed end-of the-term surveys about the program; they expressed high satisfaction with the program (*M* = 4.25 out of 5) but indicated that they would like to have more group events. They were particularly interested in future group discussions about disabilities, communication skills, and transitioning out of college. Pugliese and White (2014) also published a pilot study demonstrating that a group-based cognitive behavioral intervention could be successfully implemented with five autistic college students at Virginia Tech University. The students regarded the program as fairly helpful (*M* = 7.00 out of 10). Two students reported improvements in problem solving ability and subjective distress associated with participating in the program. Together, these initial findings from programs specifically for autistic college students suggest that a combination of-one-on-one and group-based mentorship/coaching may be helpful for autistic college students while highlighting substantial gaps in current knowledge about how to support autistic college students.

### What Skills Do Autistic College Students Need Help Developing?

Prior literature suggests that college students on the autism spectrum are particularly in need of support in three domains: social skills, self-advocacy, and executive functioning/self-regulation (e.g., Van Bergeijk et al., 2008; Schindler et al., 2015; Van Hees et al., 2015; Barnhill, 2016).

#### Social Skills

Social challenges are apparent among college students with a range of disabilities, including autism, learning disabilities (LD), and attention deficit hyperactivity disorder (ADHD; Bat-Hayim, 1997; Prevatt and Yelland, 2015; Van Hees et al., 2015). Given that social difficulties are a core aspect of the diagnostic criteria for ASD, it is likely that autistic students face particularly pronounced challenges adapting to the complex social environments they encounter in college. Indeed, many autistic college students report struggling to try “fit in” (Jones et al., 2001; Schindler et al., 2015). Some autistic college students indicate that they have developed explicit strategies that help them interact, such as scripts for different situations. Others find that the increased likelihood of sharing interests with one’s peers in college makes conversations run more smoothly than they did in high school. Nevertheless, they state that social challenges contribute to stress and cause academic difficulties (Van Hees et al., 2015).

Faculty and clinicians who work with autistic students indicate that they deviate from classroom norms by missing nonverbal cues that signal transitions between activities, interpreting sarcasm literally, standing too close to others and/or touching their belongings, talking at length about tangential topics, or remaining silent and avoiding eye contact (Gobbo and Shmulsky, 2014; Schindler et al., 2015). These social differences can lead to exclusion or even bullying (Jones et al., 2001; Gelbar et al., 2014). Social isolation may make it more difficult for autistic students to seek information from other students (MacLeod and Green, 2009) and may contribute to mental health issues (Jones et al., 2001). Indeed, anxiety and depression are commonly reported among autistic college students (Gelbar et al., 2014; Van Hees et al., 2015). Stress and social challenges may form a self-perpetuating cycle for some autistic college students; autistic adults who report the most stress also exhibit the most difficulties engaging socially (Bishop-Fitzpatrick et al., 2015).

Autistic adolescents and adults with fewer social symptoms have better relationships with peers (Orsmond et al., 2004). Therefore, effective techniques to support social skills development could reduce isolation among autistic college students. Indeed, a small body of emerging research suggests that social skills interventions for autistic adults can reduce social isolation and anxiety (Hillier et al., 2011; Spain and Blainey, 2015). Most of the limited number of specialized programs for autistic college students provide social skills supports (Gelbar et al., 2014; Barnhill, 2016). However, these supports are often neither systematically evaluated nor informed by the interests of those they are designed for. In fact, some autistic college students indicate that they are *not at all interested* in further social skills training after a lifetime of participating in such interventions (e.g., Barnhill, 2016).

Nevertheless, the majority of specialized programs for autistic college students include peer mentors and/or social coaches (Gelbar et al., 2014; Schindler et al., 2015; Barnhill, 2016). Peer mentors can provide individualized social supports to college students on the spectrum. Mentors can support students in developing social skills through discussions of techniques to use in varied social situations and by helping them to practice the techniques in role-plays; additionally, mentors can engage in campus-based activities with autistic students and provide constructive feedback afterwards (Glennon, 2001). Given that many autistic adults are not interested in training to help them act more like people who are not autistic (McLaren, 2014), programming to help autistic college students develop effective social skills should also provide them with tools to transform existing social structures, such as self-advocacy skills.

#### Self-Advocacy

Students with varied disabilities (e.g., autism, LD, and/or ADHD) often enter college without having learned how to self-advocate, or stand up for oneself and lead others (e.g., White et al., 2014). Self-advocacy skills are associated with better adaptation to college and career success among students with disabilities more generally (Rothman et al., 2008; Adams and Proctor, 2010). However, college students with disabilities are often unaware that they were protected under the Individuals with Disabilities Education Act (IDEA) prior to college, wherein schools are required to identify students who need supports, and that in college they are required to self-advocate in order to receive accommodations under the Americans with Disabilities Act (ADA).

Autistic adults may be particularly unprepared to engage in self-advocacy, as they tend to be less involved in their own transition planning relative to students with other disabilities (Fiedler and Danneker, 2007; Shogren and Plotner, 2012). Approximately 77% of autistic high school students play a very limited or no role in planning for the post-secondary school transition compared to 47% of students with intellectual disabilities and 27% of students with all other disabilities except intellectual disabilities. Only 2.6% of autistic students play a leadership role in their transition planning relative to 13.6% of students with all other disabilities except intellectual disabilities. Not surprisingly, given their relative lack of experience in transition planning, autistic college students experience difficulties with many aspects of self-advocacy, including evaluating the costs and benefits of disclosure and developing effective strategies to communicate their needs to peers and professors (Autistic Self-Advocacy Network, 2011; Van Hees et al., 2015). Given that college instructors may also be uninformed about disability laws, students on the spectrum who do not know that they must self-advocate to receive services may not receive appropriate accommodations (Pillay and Bhat, 2012).

Although the importance of teaching self-advocacy skills to autistic people has been stressed by a professor on the spectrum (Shore, 2004), parents of autistic children (Morrison et al., 2009), and researchers (Wehman et al., 2014), no published studies have examined the efficacy of self-advocacy interventions specifically designed for autistic individuals (Test et al., 2005a; Roberts et al., 2016). Therefore, a primary goal of the current research was to evaluate a self-advocacy intervention designed for autistic youth by an autistic researcher, the Integrated Self Advocacy Curriculum (Paradiz, 2009), after adapting it to suit the needs of autistic college students.

#### Study Habits/Executive Functioning Skills

Students with varied disabilities (e.g., autism, LD and/or ADHD) also face pronounced challenges developing the executive functioning/self-regulation skills needed to succeed in college (Adreon and Durocher, 2007; Parker and Boutelle, 2009; Van Hees et al., 2015). Autistic students often struggle with difficulties note taking (due to challenges identifying overarching themes and motor difficulties), completing assignments on time (due to difficulty breaking goals into sub-goals, monitoring progress toward goals, and brainstorming), organizing their study materials, and test taking (due to anxiety, motor difficulties, sensory sensitivities, and occasional processing delays). Individualized and/or group mentoring may be helpful in supporting these skills.

### Developing Supports for Autistic College Students: Benefits of Universal Design

Although students on the autism spectrum often require support to help them develop the social, self-advocacy, and executive functioning skills that they need to succeed in college, they are not alone in needing help developing these skills. Indeed, a growing body of research examines supports to help college students with the most commonly reported disabilities on college campuses, LD and/or ADHD (Newman et al., 2011), develop social, self-advocacy and executive functioning skills (Bat-Hayim, 1997; Parker and Boutelle, 2009; Brown et al., 2010; Parker et al., 2011; Harrison et al., 2012; Mytkowicz and Goss, 2012; Getzel, 2014; White et al., 2014; Prevatt and Yelland, 2015). Like the much smaller body of research evaluating supports for autistic college students (e.g., Pugliese and White, 2014; Schindler et al., 2015; Ames et al., 2016), the aforementioned evaluations of supports for college students with LD and/or ADHD were all quasi-experimental. Nevertheless, they suggest that participation in one-on-one and/or group mentoring/coaching is associated with improvements in self-advocacy, social skills, executive functioning, self-efficacy, and other domains of functioning for students with LD and/or ADHD. Although none of the participants in studies evaluating supports for college students with LD and/or ADHD were described as having an ASD, overlaps in the challenges faced by autistic students and students with LD and/or ADHD suggest that similar programming might be beneficial for neurodiverse students more generally.

Indeed, supports that are effectively designed to accommodate the needs of specific types of students (e.g., those with LD and/or ADHD), or supports that reflect the principles of Universal Design, allow students with a diverse range of strengths and weaknesses (e.g., autistic students) to learn from one another in inclusive environments where differences are valued (McGuire and Scott, 2006). Universal Design “is the design of products and environments to be usable by all people, to the greatest extent possible, without the need for adaptation or specialized design” (North Carolina State University, n.d.). Instructional materials that are consistent with the principles of Universal Design provide multiple means of representation, action and expression, and engagement (CAST, 2011). Given that autistic students exhibit highly variable patterns of academic strengths and weaknesses (Keen et al., 2016), flexible supports that are designed according to the principles of Universal Design (e.g., clear expectations, structured opportunities to interact, a constructive class culture, and consistent feedback) may be needed to help autistic students succeed in college (Burgstahler and Russo-Gleicher, 2015). Supports that students with a diverse range of disability identifications can access together may also be beneficial because many autistic college students do not identify as autistic (Newman et al., 2011) and consequently are unlikely to seek out supports designed specifically for autism.

Indeed, in the research described in this report, we initially intended to provide services *only* to autistic college students. However, we widened inclusion criteria almost immediately after realizing that many autistic students did not identify as autistic, after a number of students who did identify as autistic indicated that they had felt segregated within specialized autism programs in high school and were not interested in participating in activities that were just for autistic students, and after students who self-identified with other disabilities (some of whom appeared to have undiagnosed autism) expressed interest in participating in programming. In response to this feedback, we focused on developing and evaluating supports that were consistent with the principles of Universal Design, or supports that would be beneficial for autistic college students and their peers with other disabilities.

### Research Aims

Given that little is known about what types of supports college students with disabilities more generally feel they need and what types of supports they find effective, an important first step in developing effective and socially valid supports is to involve autistic students and students with other disabilities in the development and evaluation of such supports. Therefore, the aims of the quasi-experimental studies described in this report were:

(1) To examine neurodiverse students’ self-reported desire for guidance in developing a range of skills.
(1) To build peer-mentor-based supports that are consistent with the principles of Universal Design for autistic college students and students with other disabilities by using needs assessments collected at the beginning of each term and student feedback at the end of each term to guide program development.
(1) To evaluate self-reported benefits associated with participating in the aforementioned supports by analyzing potential changes in standardized measures from pre-test to post-test each term while attending to students’ open-ended reflections about the supports at the end of each term.

## Materials and Methods

### Structure of Mentorship Program

Students were invited to participate in weekly hour-long mentor-led group meetings with a standardized curriculum that varied each semester (see below) and/or weekly hour-long individualized, one-on-one meetings with a mentor. After completing an informed consent form, mentees filled out self-report assessments at the beginning (pre-test) and end (post-test) of each term. They received a $50 Amazon gift card for completing pre-tests and post-tests and a $10 Amazon gift card for participating in focus groups at the end of each term. Some students struggled with completing the number of forms administered and completed only a portion of them.

One-on-one mentorship was available from enrollment through finals (up to 14 weeks). Group meetings occurred over 9 or 10 weeks depending on holidays. Groups were available each day of the week and were led by a guiding mentor with the help of one or two program facilitators (doctorate or MA level). The number of mentees enrolled in each group ranged from two to nine mentees. Students who preferred not to attend group meetings were offered the group curriculum during one-on-one mentoring.

### Mentor Training

Mentors included undergraduate students, MA students, and Ph.D. students. All mentors completed an online training about autism and an hour-long in-person training designed to help them encourage their mentees to break goals into sub-goals, monitor their progress towards goals, and seek out needed resources on campus. Program facilitators were present during initial (and many subsequent) mentorship meetings to provide ongoing feedback. One-on-one mentors sent a weekly log describing each mentorship session to program facilitators and received detailed constructive feedback in response to these logs. Mentors were provided with a script for one-on-one meetings with students, but were encouraged to individualize the sessions by exploring campus activities together. For greater detail about the types of individualized activities students engage in during one-on-one mentorship, please consult (Gillespie-Lynch et al., 2017). Mentors who led groups attended three additional hours of training wherein they practiced using the group curriculum to present information in a question/answer format, which included opportunities for students to share their experiences and engage in role-plays.

### Group Curriculum

Based on prior literature and our experiences working with people on the spectrum, we identified two areas of support to address during group meetings: social skills and self-advocacy skills. During the first semester of the program, group meetings focused primarily on social skills although self-advocacy was also discussed. In response to student feedback, group meetings focused primarily on self-advocacy in the second semester of the program. Given that effective communication is essential to both social skills and self-advocacy, both semesters included components of social skills and self-advocacy.

#### Social Skills Curriculum

The initial mentorship approach that we used during the spring of 2013 was based upon the PEERS^®^ model, a social skills intervention developed by Laugeson et al. (2009). However, the intervention was altered substantially in order to be appropriate for a college student population. For example, parents were key players in the PEERS^®^ model, but were not directly involved in our social skills programming because college students are expected to become more independent of their parents. Given that college is a time when more diverse behaviors become acceptable (e.g., Robbins, 2011), we discussed how behaviors vary in how effective or ineffective they are depending on contextual factors, rather than describing behaviors as appropriate or inappropriate as they are described in the PEERS^®^ model. Curriculum topics adapted from the PEERS^®^ model included reading body language, developing conversational skills, effective use of electronic communication, respecting boundaries, and resolving disputes. Curriculum topics designed specifically for college students included self-advocacy, interview skills, and reflecting on mentorship. Consistent with the principles of Universal Design, multiple ways of engaging with the curriculum were available such as video demonstrations, role-plays and constructive group reflections on them, small group discussions, writing activities, and art activities.

#### Self-advocacy Curriculum

Our self-advocacy curriculum was adapted from the Integrated Self Advocacy Curriculum, which was created by an autistic self-advocate who is also the mother of a child on the spectrum (Paradiz, 2009). Many of the self-awareness activities and self-advocacy scripts that we used were drawn directly from the Integrated Self Advocacy Curriculum. Adaptations included inclusion of improvisational theater techniques to support development of the four domains of self-advocacy that had been identified in a literature review of prior self-advocacy interventions: self-knowledge, knowledge of rights, communication skills, and leadership skills (Test et al., 2005b). We added practice in public speaking as a core aspect of the leadership component of self-advocacy. Curriculum topics included introduction to self-advocacy and relevant laws, improvisation as a tool for self-knowledge, knowing your sensory preferences, knowing your rights under the ADA, evaluating the costs and benefits of different types of disclosure, practicing disclosure and dealing with discrimination, leadership, and public speaking. During the public speaking activity, mentees in each group worked together to develop a group PowerPoint presentation about neurodiversity, i.e., the view that disabilities represent an important aspect of human diversity that should be respected rather than cured (e.g., Kapp et al., 2013). Building from an example PowerPoint developed by mentors, mentees used their own strengths and weaknesses as examples of neurodiversity, and presented their PowerPoints to other mentors and mentees. This curriculum extended upon the principles of Universal Design employed during the social skills intervention by incorporating more multimodal activities (e.g., improvisationaltheater techniques and PowerPoint creation and presentation).

#### Fidelity

Fidelity of enactment of the curriculum for both semesters was documented with checklists of key topics covered each session. Group mentors sent weekly fidelity checks evaluating their delivery of the curriculum to program facilitators. One or two program facilitators were present at group meetings to help group leaders engage all participants as equally as possible and to document fidelity using the same checklist that group leaders used. During the second term of the program, students with disabilities also served as fidelity checkers. Fidelity of receipt was documented with worksheets mentees filled out at the end of each meeting assessing key learning objectives. One-on-one mentors sent program facilitators weekly logs of key points covered during one-on-one mentorship.

### Participants

Autistic college students and students with other disabilities who were receiving disability services at the Center for Student Accessibility (CSA) at an urban public university were invited to enroll in a mentorship program called Project REACH. Advisors at the CSA encouraged students who exhibited heightened autistic traits (irrespective of whether they self-identified as autistic and/or were identified as autistic in their paperwork) to join the mentorship program. The first and last author of this report reviewed disability documentation that students had provided to the CSA. Individualized Education Plans and Psychoeducational Reports were used to determine which students had received services for autism in high school. Some students who had documentation indicating that they had received autism services in high school did *not* self-identify as autistic in response to our demographics questionnaire while a number of students who self-identified as autistic had *not* received an educational classification of autism. When describing the participants in this research, we considered students who self-identified as autistic and/or those who had documentation of an autism classification/diagnosis to be autistic. We did not include autism in analyses given that some participants who self-identified as autistic would be unlikely to satisfy the diagnostic criteria for autism if formally evaluated while other students who did not identify as autistic would be likely to meet the diagnostic criteria for autism if evaluated.

#### Participant Characteristics: Spring Social Skills Curriculum

Twenty-eight students provided pre-test and post-test data during the spring of 2013 (18 men and 10 women; see **Table 1**). Twenty participants self-identified as White, 5 as Hispanic, 1 as Black/Native American, 1 as Muslim, and 1 as Indian. Twelve of these students self-identified as autistic (six according to educational records). One student who (selectively) self-identified as autistic although he had received an educational classification of LD had disclosed his autism diagnosis to CSA staff but did not disclose it during initial assessments for the mentorship program. However, he confided in his mentor (a disabled self-advocate whom he admired) on the last day of mentorship that he was autistic. He said that she had inspired him to educate others about disability. Until that point, he had largely avoided other students on the spectrum, whom he found annoying, but had been willing to engage with students with other disabilities. After he graduated, he obtained a job at a school for autistic children. Another student who had an educational classification of emotional disturbance rather than autism had been attending programs for autistic people from childhood into young adulthood; both she and her mother (who specialized in developmental disabilities) identified her as autistic. Another student who self-identified as autistic and whose parent also identified him as autistic had received an educational classification of LD; he exhibited black and white thinking, pronounced challenges navigating social situations (e.g., asking unknown peers if they wanted a boyfriend and becoming upset when they said no), and cognitive challenges. The other three students who self-identified as autistic but had divergent educational classifications had received educational classifications of ADHD, speech-language impairment, and a learning disability; all three of them reported and exhibited heightened autistic traits. The sixteen remaining participants self-identified with other disabilities (4 ADHD, 3 LD, 2 cerebral palsy, 1 speech-language impairment, 1 anxiety, 1 epilepsy, 1 hearing loss, 1 visual impairment, 1 an autoimmune disorder, and 1 spina bifida).

**Table 1:** Pre-test participant characteristics for spring (social skills) and fall (self-advocacy) curricula.

	Autism Ed. Class^∗^	Autism self-report	Not autism
*N* in Spring	6	6	16
% Male in Spring	83.3	66.7	56.3
% White in Spring	83.3	83.3	56.3
Age in Spring	23.0 (6.5)	23.0 (4.3)	21.3 (2.5)
SRS-A in Spring	72.7 (33.2)	61.3 (32.2)	49.8 (24.5)
Trait Anxiety in Spring	41.5 (5.8)	43.0 (14.8)	35.9 (7.4)
State Anxiety in Spring	38.2 (10.8)	38.8 (13.6)	30.4 (4.9)
*N* in Fall	12	5	13
% New to REACH in Fall	41.7	20	38.5
% Male in Fall	91.7	60	38.5
% White in Fall	66.7	80	76.9
Age in Fall	21.5 (5.5)	24.0 (4.8)	20.1 (2.7)
SRS-A in Fall	78.5 (24.9)	83.4 (53.1)	59.4 (25.8)
Trait Anxiety in Fall	44.5 (10.2)	54.2 (17.5)	39.3 (8.8)^∗∗^
State Anxiety in Fall	40.6 (11.2)	51.6 (20.5)	34.4 (8.7)^∗∗^


*Mean (SD) except where noted. ^∗^Educational Classification of autism. ^∗∗^ANOVAs revealed no group differences except heightened state and trait anxiety in those who self-reported autism relative to those who did not identify as autistic.*

#### Participant Characteristics: Fall Self-advocacy Curriculum

Of the 30 students (19 men and 11 women) who provided pre-test and post-test data during the fall of 2013, 19 had participated in the previous semester while 11 were new to the program (see **Table 1**). Twenty-two students self-identified as White, 6 as Hispanic, 1 as Black, and 1 as Mixed-Ethnicity. Seventeen students self-identified as autistic (12 according to educational records). Four of the autistic students whose self-identification did not match their educational records participated during the spring term and are described above. The student whose self-identification did not match his paperwork who enrolled in the program for the first time in the fall had an educational classification of speech-language impairment. He struggled socially due to a pronounced disinterest in small talk and a tendency to consistently wish to engage people in deep discussions about a few topics that were of intense interest to him (e.g., religion, morality, and identity). Thirteen students reported other disabilities (8 learning disability, 2 cerebral palsy, 1 emotional disturbance, 1 Tourette’s syndrome, and 1 hearing loss).

### Measures

#### Needs Assessments

Students rated the perceived importance of receiving guidance on 39 skills, derived from prior literature, using a 5-point scale from 1 (very unimportant) to 5 (very important). Skills included social skills (e.g., “Maintaining friendships”), self-advocacy skills (e.g., “Disclosing one’s diagnosis”), self-regulation skills (e.g., “Coping with change”), job skills (e.g., “Developing interview skills”), and academic skills (e.g., “Meeting educational goals”). Students were also provided with an open-ended opportunity to indicate things they felt were important that were not assessed.

Needs assessments were distributed to all students who expressed interest in the mentorship program by attending a pizza party prior to the first term of the mentorship program in the spring of 2013. Needs assessments were also distributed during pre-test assessments prior to the second semester of the mentorship program in the fall of 2013. Needs assessments were used to guide the selection of specific topics to cover each term; needs that participants rated as highly important at the beginning of each term were included in that semester’s curriculum.

#### Demographics Questionnaire

Students were asked to indicate if they self-identified as autistic, and their age, gender, and race.

### Multidimensional Scale of Perceived Social Support

This 12-item measure assesses perceived social support in three domains: family, friends, and significant others (Zimet et al., 1988). Participants rated their agreement with items like “I can talk about my problems with my friends” on a scale from 1 (very strongly disagree) to 7 (very strongly agree). The measure has been shown to have moderate construct validity, as assessed by negative correlations between perceived social support and depression and anxiety. Its internal consistency was good at baseline in the current sample (Spring α = 0.85; Fall α = 0.96). However, we did not expect the mentorship program to alter familial relationships. In addition, some students chose not to respond to questions assessing support from a significant other. Therefore, we examined both changes in overall social support and changes in social support from friends in particular.

#### Social Responsiveness Scale-A

The Social Responsiveness Scale-A (SRS-A; Constantino and Gruber, 2012) is a self-report measure of autism symptoms consisting of five subscales: Social Awareness, Social Cognition, Social Communication, Social Motivation, and Restricted Interests and Repetitive Behaviors. Higher scores indicate heightened levels of autistic traits. Prior research has shown the SRS to be internally consistent with strong construct validity in both the general population and clinical samples (Constantino and Gruber, 2012; Wigham et al., 2012). The internal consistency for the SRS-A was excellent at pre-test in the current sample (Spring α = 0.94; Fall α = 0.96).

#### Spielberger State-Trait Anxiety Inventory

The 40-item State-Trait Anxiety Inventory (Spielberger et al., 1983) consists of two 20-item scales that assess trait anxiety (how anxious people generally feel) and state anxiety (how anxious people feel at the moment). Cronbach’s alpha revealed high internal consistency at pre-test (Spring α = 0.93; Fall α = 0.95).

#### Student Self-report of Academic Self-efficacy

This measure was used to examine students’ belief in their academic abilities (Hoover-Dempsey and Sandler, 2005). They were asked to rate their perceived ability to learn the things taught in school, to do even the hardest homework if they try, and to figure out difficult homework on a 4-point scale from 1 (not true) to 4 (very true). At pre-testing, this measure had acceptable internal consistency (Spring α = 0.77; Fall α = 0.77).

#### Focus Groups

Students were asked a series of structured questions during focus groups at the end of each semester, such as “What was your favorite/least favorite part of (the mentorship program) this term?” and “What are the ways, if any, that participating in (the mentorship program) this term has helped you develop social/academic/self-advocacy skills.” Consistent with the inclusive structure of the program and the need to obtain feedback from all involved, mentors and mentees were invited to participate in focus groups together.

The following measures were added in response to feedback from students during the focus groups at the end of the first term of the program (Spring 2013 social skills curriculum) and were only obtained during the second term (Fall 2013 self-advocacy curriculum).

#### Self-advocacy Inventory

Given the lack of standardized self-advocacy measures when we conducted this study, we developed a self-advocacy questionnaire based upon the conceptual framework for self-advocacy developed by Test et al. (2005b). Closed-ended questions assessed knowledge of self (e.g., “I know my own strengths”), knowledge of rights (e.g., “The Americans with Disabilities Act is the law that is most relevant to college students with disabilities”), communication skills (e.g., “I am skilled at communicating one-on-one with others”), and leadership skills (e.g., “I am a skilled leader”). Students indicated their agreement to 12 such statements on a scale from 1 (strongly disagree) to 5 (strongly agree). The measure exhibited acceptable internal consistency at baseline (Fall α = 0.80).

#### Open-ended Self-advocacy Definitions

We also asked participants to provide a written response to the open-ended question “What is self-advocacy in your own words?” The first author used a thematic approach (Braun and Clarke, 2006) guided by Test et al. (2005b) conceptual definition of self-advocacy to develop a coding scheme for participants’ responses to this question. Four coding categories identified elements of each response that were consistent with Test’s conceptual framework (examples of student responses are provided in parentheses after each coding category): self-knowledge (e.g., “Self-advocacy is the ability to speak out for yourself in regards to *your abilities and disabilities*”), knowledge of rights (e.g., “Self-advocacy means to ask for *accommodations that are reasonable*”), communication skills (e.g., “*Explaining* one’s disability”), and leadership (e.g., “Self-advocacy is when a *person is a leader* and becomes their own person”). Four coding categories were developed that diverged from Test’s conceptual framework: self-esteem (e.g., “To me self-advocacy is *self-confidence*”), self-reliance (e.g., “How you *help yourself*, finding your own references”), “don’t know”/blank, or other (responses fitting none of the aforementioned codes). Responses were qualitatively coded by two independent coders who achieved reliabilities of 80% or greater across coding categories for 20% of all responses. A self-advocacy knowledge score, ranging from 0 to 4, was derived by summing the number of elements of Test’s definition of self-advocacy that were present in each response.

#### End of Term Written Evaluations

Given that not all students were comfortable expressing their viewpoints during focus groups, we embedded open-ended written opportunities to evaluate the program in post-testing at the end of the fall term with the questions “What was your favorite part of Project REACH this term?” and “What was your least favorite part of Project REACH this term?”

## Results

### Analytic Approach

We utilized descriptive analyses to identify the five needs that students ranked most highly on average at the beginning of each term. Descriptive analysis of the data indicated that the summed totals derived from most of the surveys administered to participants at pre-test and post-test did not exhibit excessive kurtosis and/or skew. Therefore, we used general linear models (repeated measures ANOVAs) to evaluate potential changes from pre-test to post-test in most measures. We included prior experience in the mentorship program as a between-subjects variable in models conducted with data collected in the fall. However, descriptive analyses indicated that academic self-efficacy, perceived social support and self-advocacy knowledge scores (derived from qualitative coding of open-ended responses) exhibited excessive kurtosis and/or skew. Therefore, we used non-parametric tests (Wilcoxon signed-rank tests) to evaluate potential changes in these measures from pre-test to post-test. A two-tailed significance level (α ≤ 0.05) was used for all analyses. Given that many studies (including the current study) are underpowered, Thompson (1998) recommended that researchers report effect sizes for both statistically significant and non-significant findings. Therefore, we provide effect sizes for both significant and non-significant results.

### Needs Assessments Prior to the Spring Social Skills Curriculum

Eighteen students (12 of whom were autistic according to self-report and/or educational classification) completed needs assessments during an informational meeting about the mentorship program prior to pre-testing for the spring term. Several of these students did not go on to participate in the program. The five highest ranked needs were maintaining friendships (*M* = 4.22, *SD* = 1.31), forming friendships (*M* = 4.17, *SD* = 1.34), listening to others (*M* = 4.06, *SD* = 1.25), developing work skills (*M* = 4.06, *SD* = 1.51), and picking a career (*M* = 4.06, *SD* = 1.35).

### Pre-test to Post-test Comparisons: Spring Social Skills Curriculum

Repeated measures ANOVAs were used to examine changes from pre-test to post-test that were associated with participation in the social skills curriculum. Autism symptoms (SRS-A scores) decreased from pre-test (*M* = 58.24, *SD* = 28.95) to post-test [*M* = 49.30, *SD* = 30.33; *F*(1,22) = 7.23, *p* = 0.01; η^2^ = 0.25]. Trait anxiety decreased from pre-test (*M* = 38.68, *SD* = 9.14) to post-test [*M* = 35.56, *SD* = 9.92; *F*(1,24) = 7.68, *p* = 0.01; η^2^ = 24] whereas reductions in state anxiety were not observed (*p* = 0.22; η^2^ = 0.06). Analyses of potential changes in overall perceived social support (*p* = 0.78; *r* = 0.04), perceived social support from friends (*p* = 0.06; *r* = 0.27; note this is a trend toward *reduced* perceived social support from friends), and academic self-efficacy (*p* = 0.43; *r* = 0.11) revealed no significant changes associated with participating in the social skills intervention.

### End of Term Focus Groups: Spring Social Skills Curriculum

Ten students and three mentors participated in three optional focus groups at the end of the spring term. While some students reported social benefits of the social skills training, such as helping a student do a “little bit more of the social going out” others stated that they hadn’t needed social skills help. For example, a student said, “I came into the program when I was already well in social skills.”

Interestingly, given that self-advocacy was a topic of the social skills curriculum, but not the primary focus, a number of students described self-advocacy related benefits of the program. For example, one student said it helped her in “taking the initiative to do things on my own instead of people telling me what to do (such as)… talking to those of higher authority… I think the biggest (idea) …we can take away from (the mentorship program) this semester is in general is not to be ashamed or embarrassed about our disability. It’s only a part of who you are.” Another student said, “I used to be ashamed. I didn’t like talking about it or anything. From (the mentorship program), I got confidence.” Another student’s agreement with this point suggested that greater support in developing self-advocacy was needed: “My parents would say ‘don’t say you have what you have?’ But then that also comes into that we need to learn. When do you talk about your disability? When do you disclose and when do you leave it as it is?”

In further support of the idea that the students needed more guidance in developing self-advocacy skills, a number of students described relying on others to advocate for them. For example, one student described the process of obtaining accommodations as “(my advisor) starts writing letters to the teachers and talks to them about myself and what I have.” Another student said, “As for speaking to professors about stuff like that if they have a problem with it, I usually just get (name of the director of the Center for Student Accessibility) on it.” Another student described asking her advisor to “self-advocate for me.” Given this evidence that students would benefit from self-advocacy instruction, we focused the Project REACH curriculum on self-advocacy in the following semester.

### Needs Assessments Prior to the Fall Self-advocacy Curriculum

Twenty-seven students (15 of whom were autistic) completed needs assessments. The highest ranked needs were communicating with teachers (*M* = 4.48, *SD* = 0.92), building a resume (*M* = 4.48, *SD* = 1.01), dealing with social anxiety (*M* = 4.44, *SD* = 0.97), meeting educational goals (*M* = 4.44, *SD* = 1.19), and developing work skills (*M* = 4.44, *SD* = 1.08).

### Pre-test to Post-test Comparisons: Fall Self-advocacy Curriculum

In the fall, repeated measures analyses were conducted with prior experience in Project REACH as a between-subjects factor in all parametric analyses. No interactions between prior experience and changes in self-reported characteristics were observed. Although not a focus of our analytic approach, students who had been in Project REACH in the spring reported lower autism symptoms, state and trait anxiety and higher perceived self-advocacy skills (the summed total on closed-ended self-advocacy items) and social support from friends than students who were new to the program in the fall (*p*s < 0.05).

Repeated measures analyses revealed *no changes* from pre-test to post-test in self-reported autism symptoms (*p* = 0.24; η^2^ = 0.05), state anxiety (*p* = 0.44; η^2^ = 0.02), trait anxiety (*p* = 0.24; η^2^ = 0.05), and overall perceived social support (*p* = 0.42; *r* = 0.11) associated with participating in the fall self-advocacy curriculum. However, perceived social support from friends increased from pre-test (*M* = 20.92, *SD* = 6.80) to post-test (*M* = 22.38, *SD* = 5.33; *Z* = –2.34, *p* = 0.02; *r* = 0.32). Academic self-efficacy also increased from pre-test (*M* = 8.79, *SD* = 1.89) to post-test (*M* = 9.75, *SD* = 2.03; *Z* = –2.893, *p* = 0.004, *r* = 0.39). Changes in scores on the closed-ended self-advocacy questionnaire were *not* observed (*p* = 0.61; η^2^ = 0.01). In contrast, self-advocacy knowledge scores, or the number of elements of Test’s (2005) definition of self-advocacy that were present in students’ open-ended definitions of self-advocacy, increased from pre-test (*M* = 0.64, *SD* = 0.83) to post-test (*M* = 1.29, *SD* = 0.94; *Z* = –3.07, *p* = 0.002, *r* = 0.41).

### End of Term Written Evaluations: Fall Self-advocacy Curriculum

Twenty-one mentees responded to the prompt asking them to write what they liked most and least about the mentorship program during post-tests. Seven students specified that they enjoyed learning about self-advocacy. For example, one student wrote, “My favorite part about Project REACH this semester was probably the aspect of us learning about self-advocacy. I felt accepted to the fact that people wanted to hear my specific strengths and weaknesses.” Another student wrote, “My favorite part about Project REACH this term is learning about self-advocacy and hearing about students to tell their story to everyone.” Two students particularly enjoyed the public speaking component of the self-advocacy training. For example, one student wrote, “My favorite part about Project REACH is when we made slides about us.”

Eight students indicated that they particularly enjoyed opportunities for social interaction provided by the mentorship program. For example, one student wrote that his favorite part of the program was “Socializing with nice people and hanging with my friend (while he was here) and learning from a great mentor.” Another student indicated that he liked to “Meet more people like me.” Another student wrote that she liked “Meeting new people and to learn new things.” Another student wrote that he liked to “Socialize with everyone and discuss our problems.”

Six students provided general responses to the question that were not clearly related to either self-advocacy or social interactions. For example, one student wrote that he enjoyed “The group process meeting and the exercises” while another enjoyed “Being involved in the groups while observing at the same time.” Two students indicated that they preferred “One-on-one mentorships.”

Eight students specified that they had no least favorite part of Project REACH. For example, one student wrote, “I enjoyed being a part of the program every bit.” However, four students indicated that they disliked completing pre-test and post-test assessments. For example, one student wrote that he particularly disliked “Answering the same exact question at the start and end of this.” Three students indicated that group meetings were sometimes anxiety provoking. For example, one student wrote that he did not enjoy “Some group sessions which brought a higher-than-normal level of uncomfortability, especially around unfamiliar people.” One student wrote that he particularly did not like “Presenting a speech.” Two students indicated that group meetings were sometimes dull. For example, one student wrote “That it can get a little boring sometimes. Suggestion–more activities.” Three students stated that they did not like other students’ behaviors during groups. For example, one student wrote that she did not enjoy “When one person talks too much.”

### End of Term Focus Groups: Fall Self-advocacy Curriculum

Twelve mentees and four mentors participated in two optional focus groups at the end of the fall term. Students reported that they had enjoyed learning more about self-advocacy. For example, one student said “Talking about self-advocacy…was so important to me… in relation to my own history of being advocated for… for a long time our voices have been hushed… only now are they letting our voices be heard.” Another student said, “The one thing that incoming freshman should know, is you have to advocate for yourself…it’s all on you.” Another student agreed, “Because when you get into college you need to be your own person, especially with a disability you have to let them know you need certain… accommodations.” Another student described the importance of educating professors about self-advocacy stating that many “are not even aware of the ADA.”

Nevertheless, many students reported that their favorite part of mentorship remained its social aspects. For example, one student said, “My favorite part is meeting new people, which is my favorite part about anything.” Another student said, “I think my favorite part was just being able to meet so many people like me.” Another student said that she’d enjoyed her second term of mentorship because “I’ve kind of learned to come out my shell, if I ever was in a shell.” Another student reported that the mentorship program had “helped me to socialize with other people. It’s also taught me to be patient with those that are both patient and impatient with me.” Another student said that the mentorship program gave “me a chance to socialize. ‘Cause I’m not going to lie, I don’t like people; I don’t like talking to people… You guys are different though. ‘Cause it’s like, I have more of a tolerance for people here.”

## Discussion

Students’ high rankings of the importance of receiving guidance and opportunities to improve their social skills is consistent with an emerging body of literature demonstrating that social skills training can be beneficial for autistic adolescents and young adults (e.g., Laugeson et al., 2009; Gantman et al., 2012; Yoo et al., 2014; Spain and Blainey, 2015). Extending an experimental evaluation of the PEERS^®^ model (which our social skills training was adapted from, in order to support college students in particular), our quasi-experimental results suggest that social skills training is associated with decreases in autism symptoms and trait anxiety among neurodiverse students. Although increases in social interactions were observed among participants in the PEERS^®^ training, increased social support from friends was *not* observed following our social skills training. This difference may be attributable to the lesser density of social skills training in our program as well as the lack of parental involvement, or the higher levels of social support at baseline among our participants, in comparison to participants in the PEERS^®^ program.

Our findings build upon a small body of work, suggesting that social skills training can be helpful for autistic adults (Spain and Blainey, 2015) and college students with other disabilities (e.g., Bat-Hayim, 1997) by suggesting that a Universal Design approach to social skills training is beneficial for college students with diverse disabilities, including autism. However, a number of students with and without autism who participated in our mentorship program indicated that they had not needed further supports to develop social skills prior to participating. This lack of interest in social skills training among some autistic college students is consistent with reports from other programs for college students on the spectrum (Barnhill, 2016). Social skills interventions for autistic individuals often focus on teaching people with autism how to interact more like their “typical” peers (e.g., Laugeson et al., 2009). However, many individuals on the spectrum may not view autism as something they wish to mask by learning to appear more “normal”, but rather view it as an important aspect of their identity (Kapp et al., 2013). Although our social skills training was designed to emphasize flexibility in social interactions, autistic college students may have extended experience participating in social skills interventions and opinions about its utility. The social validity of supports for neurodiverse adults is likely to be maximized by adapting services to their concerns in order to help them navigate toward environments where their quirks are appreciated, including opportunities to engage with neurodiverse peers.

Self-advocacy may be a particularly relevant topic for college students with disabilities given pronounced changes in how accommodations are obtained in college relative to high school. Indeed, students with and without autism expressed strong interest in self-advocacy during focus groups at the end of each term. Their interest in learning more about self-advocacy was less pronounced than their interest in social and job-related skills during needs assessments at the beginning of each term (the perceived importance of learning more about “self-advocacy: disability disclosure” was 3.14 out of 5 at the beginning of the spring term and 4.22 out of 5 at the beginning of the fall term), which may reflect a relative lack of knowledge at the beginning of each term about what self-advocacy is. Indeed, students’ open-ended definitions of self-advocacy at the beginning of the fall term contained an average of less than 1 out of 4 of the elements defined as essential to self-advocacy by Test et al. (2005b).

Surprisingly, participation in the self-advocacy training was *not* associated with increased self-advocacy as assessed with the self-advocacy questionnaire that we developed. However, the measure we developed assessed students’ *beliefs* about their self-advocacy skills rather than the knowledge and skills needed to *engage* in self-advocacy. Indeed, significant improvements in students’ *ability* to define self-advocacy were observed after participation in the program. This pattern of findings suggests that self-advocacy assessments should focus on direct assessments of knowledge (e.g., fact based rather than opinion based questionnaires) and skills (e.g., role-plays). In a recent quasi-experimental evaluation of a promising hybrid (online and in-person) self-advocacy curriculum conducted with college students with varied disabilities (none of whom were described as autistic), White et al. (2014) used a fact-based questionnaire and role-plays. Although White and colleagues’ role-plays should be improved upon, as they were not counterbalanced and participants were given a sheet to use only during post-test role-plays that listed target skills to demonstrate, similar types of role-plays should be incorporated in future research evaluating services for neurodiverse college students.

Unlike participation in the social skills training, participation in the self-advocacy training was associated with increased perceived social support from friends and heightened academic self-efficacy, but was not associated with reductions in autism symptoms or anxiety. Specific benefits associated with each type of training suggest that a combination of social skills and self-advocacy training is likely to be most beneficial for autistic students and their peers with other disabilities. Given students’ interest in developing work-related skills, such programming should highlight how social and self-advocacy skills can be applied when seeking a job and in the workplace.

However, the specific curricular focus of programming for autistic college students and other students with disabilities may be less important than the opportunities to engage with peers that group programming provides. Indeed, it is impossible to rule out the possibility that apparent benefits of programming designed for neurodiverse students reported in the current study and in prior literature have been *entirely attributable* to the opportunities to engage with peers that such programming provides as *all* (to the best of our knowledge) of the extant published research examining supports for autistic college students and college students with other disabilities has been quasi-experimental (e.g., Bat-Hayim, 1997; Parker et al., 2011; Harrison et al., 2012; Mytkowicz and Goss, 2012; Getzel, 2014; Pugliese and White, 2014; White et al., 2014; Prevatt and Yelland, 2015; Schindler et al., 2015; Ames et al., 2016). Indeed, many students in the current study reported that their favorite part of the mentorship program remained the opportunities it provided to socialize with peers in an environment where differences were respected, irrespective of the curricular topic. Nevertheless, engaging students with a curriculum that provides information about self-advocacy (which was included in both semesters of the program though it was the primary focus only in the second term) is likely to encourage a climate of acceptance (which is a central aspect of Universal Design).

## Limitations and Future Directions

A key limitation of our research is that we were unable to verify autism classifications with clinical diagnoses informed by gold standard measures such as the Autism Diagnostic Observation Schedule. It is likely that some students who were classified as autistic according to self-report and/or educational documents would not meet diagnostic criteria for autism while others who were identified as not autistic may actually meet criteria for autism. Prior work with adults has often relied upon educational classifications of autism (e.g., Newman et al., 2011; Wei et al., 2013; Shattuck et al., 2014). These studies have often relied on longitudinal data sets rather than direct dialog with participants. Our experiences interacting directly with college students on the spectrum suggest that some families and/or school personnel may preferentially choose educational classifications other than autism. The decision not to identify as autistic may arise from multiple factors including internalized stigma towards autism. Based on our discussions with parents, teachers, and autistic young adults, we believe that some families may feel that an educational classification of autism can limit their children’s opportunities and selectively choose other classifications. In addition, school personnel may shy away from supporting a classification of autism due to the possible costs of supporting a student who has been identified as autistic. Future research should examine factors, such as perceived stigma and the types of supports different educational classifications entitle people to, that may influence which educational determination families and educators choose, as well as variations in the number of students with confirmed diagnoses of ASD who receive accommodations for autism across different geographical regions and at different developmental stages.

A second key limitation of our research is our reliance on a quasi-experimental design wherein some students participated in both sessions of the mentorship program and others did not. It is not possible to make causal attributions about effects of mentorship with this type of design. Previous peer-reviewed work examining supports for autistic college students and students with other disabilities has also utilized pre-test/post-test designs (often with a similarly small number of students). These flaws in design may arise from complications associated with random assignment of college students, such as the need to adapt programming to their busy schedules. In order to conduct systematic randomized evaluations of programming for neurodiverse college students, researchers from varied institutions would need to obtain shared funding in order to collaboratively randomize students to shared interventions. Random assignment to different types of interventions, rather than to wait-list control groups, would allow researchers to evaluate if benefits of programming are attributable to specific aspects of a given curriculum or to structured opportunities to engage with peers more generally. Such work should include standardized measures from multiple informants, behavioral measures, and structured interviews in order to assess self-advocacy and social skills more effectively than we did by relying on the self-report of students. A randomized comparison of social skills and self-advocacy interventions in adolescence and adulthood could be informative for highlighting potential developmental changes in the effects of different types of interventions.

## Conclusion

These findings have clear practical implications. They support previous literature by demonstrating that autistic college students may face challenges navigating the college environment, including social difficulties, heightened anxiety, problems with self-advocacy, and a sense of being stigmatized. However, these challenges are not specific to autism. Autistic students and students with other disabilities expressed shared interest in helping to develop their social, employment-related, academic, and self-advocacy skills.

Not only do students on the spectrum have support needs that are compatible with those of other types of students, some autistic college students may avoid supports that are just for autistic people. Indeed, a few autistic students told us that they found the idea of a program that was just for autistic students discriminatory. In addition, a number of participants in our mentorship program who had educational classifications of autism did not consistently self-identify as autistic. Students who do not identify as autistic, who may represent a sizable portion of the population of autistic college students (e.g., Newman et al., 2011), may be unlikely to seek out autism-specific services as engaging in autism-specific services would constitute a form of disclosure. Not only may supports that are designed for autistic students *and* students with other disabilities be more attractive to some college students on the spectrum, experience engaging with neurodiverse others may be beneficial for students more generally by demonstrating that varied ways of existing are equally valid while offering different types of models to learn from.

We found that curriculum initially developed to address challenges college students on the autism spectrum face was relevant to students with a range of disabilities. Thus, a primary recommendation of our work is that services for autistic college students should reflect the principles of Universal Design and should include a variety of other students, including those with other disabilities. Peer mentorship models wherein students with and without disabilities serve as mentors may support the development of programming that conforms to the principles of Universal Design as they allow mentees and mentors to shape their interactions and programming together.

Inclusion of autistic students in program evaluation and modification is also likely to support the creation of a respectful environment and empowered community. Students expressed a great deal of enjoyment of their ability to influence the development of the program by providing their recommendations during focus groups and as fidelity checkers. Admittedly, their level of involvement during the two semesters of the mentorship program described in this report remained limited; while they helped evaluate and modify the structure and focus of the program, they did not play a direct role in research. However, in subsequent semesters of the mentorship program, autistic students and those with other disabilities have gone on to become mentors themselves, have developed their own research questions about the program in the context of credit-bearing independent studies; they have also synthesized other students’ evaluations of the program and prior literature to generate recommendations for and act as mentors in summer transition programs for incoming autistic college students. Perhaps the most essential recommendation arising from this work is that autistic college students and their peers should be increasingly empowered participants in research designed to support them.

## Ethics Statement

This study was carried out in accordance with the recommendations of the Institutional Review Board of the College of Staten Island, CUNY with written informed consent from all subjects. All subjects gave written informed consent in accordance with the Declaration of Helsinki. The protocol was approved by the College of Staten Islands Institutional Review Board.

## Author Contributions

KG-L led the development and evaluation of the mentorship program and played the primary role in analyses and writing this manuscript. DB played a leading role in data entry and quality checking, contributed to data analyses and contributed to the writing of the manuscript. AD contributed substantially to the development of the mentorship program, recruitment, support for and assessment of the mentorship program. VW contributed substantially to data entry and to the development of the mentorship curriculum (as a mentor). PB contributed to the initial design of the mentorship program and the writing of the manuscript. JD played a guiding role in recruitment, helping to design the mentorship program and verification of diagnoses.

## Conflict of Interest Statement

The authors declare that the research was conducted in the absence of any commercial or financial relationships that could be construed as a potential conflict of interest.

## Footnotes

1 The term “autistic person” is preferred throughout this report rather than “person with autism” in order to respect the preferences of autistic people who often prefer “identity first” terms over “person first” terms (e.g., Kapp et al., 2013; Kenny et al., 2016).

## References

[B1] Adams K. S. Proctor B. E. (2010). Adaptation to college for students with and without disabilities: group differences and predictors. *J. Postsecond. Educ. Disabil.* 22 166–184.

[B2] Adreon D. Durocher J. S. (2007). Evaluating the college transition needs of individuals with high-functioning autism spectrum disorders. *Interv. Sch. Clin.* 42 271–279. 10.1177/10534512070420050201

[B3] Ames M. E. McMorris C. A. Alli L. N. Bebko J. M. (2016). Overview and evaluation of a mentorship program for university students with ASD. *Focus Autism Other Dev. Disabil.* 31 27–36. 10.1177/1088357615583465

[B4] Autistic Self-Advocacy Network (2011). *Navigating College: A Handbook on Self Advocacy Written for Autistic Students from Autistic Adults*. Available at: http://www.navigatingcollege.org/download.php

[B5] Barnhill G. P. (2016). Supporting students with asperger syndrome on college campuses: current practices. *Focus Autism Other Dev. Disabil.* 31 3–15. 10.1177/1088357614523121

[B6] Bat-Hayim M. (1997). Learning to learn: learning therapy in a college classroom. *Ann. Dyslexia* 47 203–238. 10.1007/s11881-997-0027-5

[B7] Bebko J. M. Schroeder J. H. Ames M. E. (2011). *A Mentoring Program for Students with Asperger and ASDs.* Toronto, ON: York University.

[B8] Bishop-Fitzpatrick L. Mazefsky C. A. Minshew N. J. Eack S. M. (2015). The relationship between stress and social functioning in adults with Autism Spectrum Disorder and without intellectual disability. *Autism Res.* 8 164–173. 10.1002/aur.1433 25524571 PMC4412754

[B9] Bishop-Fitzpatrick L. Minshew N. J. Eack S. M. (2013). A systematic review of psychosocial interventions for adults with autism spectrum disorders. *J. Autism Dev. Disord.* 43 687–694. 10.1007/s10803-012-1615-8 22825929 PMC3508309

[B10] Braun V. Clarke V. (2006). Using thematic analysis in psychology. *Qual. Res. Psychol.* 3 77–101. 10.1191/1478088706qp063oa

[B11] Brown S. E. Takahashi K. Roberts K. D. (2010). Mentoring individuals with disabilities in postsecondary education: a review of the literature. *J. Postsecond. Educ. Disabil.* 23 98–111.

[B12] Burgstahler S. Russo-Gleicher R. (2015). Applying universal design to address the needs of postsecondary students on the autism spectrum. *J. Postsecond. Educ. Disabil.* 28 199–212.

[B13] Cai R. Y. Richdale A. L. (2016). Educational experiences and needs of higher education students with autism spectrum disorder. *J. Autism Dev. Disord.* 46 31–41. 10.1007/s10803-015-2535-1 26216381

[B14] CAST (2011). *Universal Design for Learning Guidelines version 2.0*. Wakefield, MA: Author.

[B15] Cederlund M. Hagberg B. Billstedt E. Gillberg I. C. Gillberg C. (2008). Asperger syndrome and autism: a comparative longitudinal follow-up study more than 5 years after original diagnosis. *J. Autism Dev. Disord.* 38 72–85. 10.1007/s10803-007-0364-6 17340200

[B16] Cimera R. E. Cowan R. J. (2009). The costs of services and employment outcomes achieved by adults with autism in the US. *Autism* 13 285–302. 10.1177/1362361309103791 19369389

[B17] Constantino J. N. Gruber C. P. (2012). *Social Responsiveness Scale-2*. Los Angeles, CA: Western Psychology Services.

[B18] Fiedler C. R. Danneker J. E. (2007). Self-advocacy instruction: bridging the research-to-practice gap. *Focus Except. Child.* 39 1–20. 10.1093/deafed/enq038

[B19] Gantman A. Kapp S. K. Orenski K. Laugeson E. A. (2012). Social skills training for young adults with high-functioning autism spectrum disorders: a randomized controlled pilot study. *J. Autism Dev. Disord.* 42 1094–1103. 10.1007/s10803-011-1350-6 21915740

[B20] Gelbar N. W. Smith I. Reichow B. (2014). Systematic review of articles describing experience and supports of individuals with autism enrolled in college and university programs. *J. Autism Dev. Disord.* 44 2593–2601. 10.1007/s10803-014-2135-5 24816943

[B21] Getzel E. E. (2014). Fostering self-determination in higher education: identifying evidence-based practices. *J. Postsecond. Educ. Disabil.* 27 381–386.

[B22] Gillespie-Lynch K. DeNigris D. Cheriyan B. Massa A. Wong V. Kostikas C. (2017). “Fostering effective teaching using strategies developed by peer mentors for autistic and non-autistic undergraduates,” in *How We Teach Now: The GSTA Guide to Student-Centered Teaching*, eds Obeid R. Schwartz A. Shane-Simpson C. Brooks P. J. (Washington, DC: Society for the Teaching of Psychology).

[B23] Glennon T. J. (2001). The stress of the university experience for students with Asperger syndrome. *Work* 17 183–190. 12441598

[B24] Gobbo K. Shmulsky S. (2014). Faculty experience with college students with autism spectrum disorders: a qualitative study of challenges and solutions. *Focus Autism Other Dev. Disabil.* 29 13–22. 10.1177/1088357613504989

[B25] Harrison A. G. Areepattamannil S. Freeman J. (2012). Effects of the learning opportunities task force (LOTF) programs on postsecondary students with learning disabilities. *Except. Educ. Int.* 22 55–69.

[B26] Hendricks D. (2010). Employment and adults with autism spectrum disorders: challenges and strategies for success. *J. Vocat. Rehabil.* 32 125–134.

[B27] Hillier A. Galizzi M. (2014). Employment outcomes for young adults with autism spectrum disorders. *Rev. Disabil. Stud. Int. J.* 10 69–82.

[B28] Hillier A. J. Fish T. Siegel J. H. Beversdorf D. Q. (2011). Social and vocational skills training reduces self-reported anxiety and depression among young adults on the autism spectrum. *J. Dev. Phys. Disabil.* 23 267–276. 10.1007/s10882-011-9226-4

[B29] Hoover-Dempsey K. V. Sandler H. M. (2005). Final performance report for OERI Grant # R305T010673: the social context of parental involvement: a path to enhanced achievement. *Paper Presented to Project Monitor, Institute of Education Sciences, U.S. Department of Education, March 22, 2005* Washington, DC.

[B30] Howlin P. Goode S. Hutton J. Rutter M. (2004). Adult outcome for children with autism. *J. Child Psychol. Psychiatry* 45 212–229. 10.1111/j.1469-7610.2004.00215.x 14982237

[B31] Jones R. S. P. Zahl A. Huws J. C. (2001). First-hand accounts of emotional experiences in autism: a qualitative analysis. *Disabil. Soc.* 16 393–401. 10.1080/09687590120045950

[B32] Kapp S. Gillespie-Lynch K. Sherman L. Hutman T. (2013). Deficit, difference or both? Autism and neurodiversity. *Dev. Psychol.* 49 59–71. 10.1037/a0028353 22545843

[B33] Kapp S. K. Gantman A. Laugeson E. A. (2011). “Chapter 25–Transition to adulthood for high-functioning individuals with autism spectrum disorders,” in *A Comprehensive Book on Autism Spectrum Disorders*, ed. Mohammadi M.-R. (Rijeka: InTech).

[B34] Keen D. Webster A. Ridley G. (2016). How well are children with autism spectrum disorder doing academically at school? An overview of the literature. *Autism* 20 276–294. 10.1177/1362361315580962 25948598

[B35] Kenny L. Hattersley C. Molins B. Buckley C. Povey C. Pellicano E. (2016). Which terms should be used to describe autism? Perspectives from the UK autism community. *Autism* 20 442–462.0. 10.1177/1362361315588200 26134030

[B36] Laugeson E. A. Frankel F. Mogil C. Dillon A. R. (2009). Parent-assisted social skills training to improve friendships in teens with autism spectrum disorders. *J. Autism Dev. Disord.* 39 596–606. 10.1007/s10803-008-0664-5 19015968

[B37] MacLeod A. Green S. (2009). Beyond the books: case study of a collaborative and holistic support model for university students with Asperger syndrome. *Stud. High. Educ.* 34 631–646. 10.1080/03075070802590643

[B38] McGuire J. M. Scott S. S. (2006). Universal design for instruction: extending the universal design paradigm to college instruction. *J. Postsecond. Educ. Disabil.* 19 124–134.

[B39] McLaren K. R. (2014). *Interrogating Normal: Autism Social Skills Training at the Margins of a Social Fiction*. Doctoral dissertation, Sonoma State University, Rohnert Park, CA.

[B40] Morrison J. Q. Sansosti F. J. Hadley W. M. (2009). Parent perceptions of the anticipated needs expectations for support for their college-bound students with Asperger’s Syndrome. *J. Postsecond. Educ. Disabil.* 22 78–87.

[B41] Mytkowicz P. Goss D. (2012). Students’ perceptions of a postsecondary LD/ADHD support program. *J. Postsecond. Educ. Disabil.* 25 345–361.

[B42] Newman L. Wagner M. Knokey A. M. Marder C. Nagle K. Shaver D. (2011). *The Post-High School Outcomes of Young Adults with Disabilities up to 8 Years After High School: A Report from the National Longitudinal Transition Study-2 (NLTS2). NCSER 2011-3005*. Menlo Park, CA: SRI International.

[B43] North Carolina State University (n.d.). *The Principles of Universal Design*. Raleigh, NC: Center for Universal Design, North Carolina State University.

[B44] Orsmond G. I. Krauss M. W. Seltzer M. M. (2004). Peer relationships and social and recreational activities among adolescents and adults with autism. *J. Autism Dev. Disord.* 34 245–256. 10.1023/B:JADD.0000029547.96610.df 15264493

[B45] Paradiz V. (2009). *The Integrated Self-Advocacy ISA Curriculum: A Program for Emerging Self-Advocates with Autism Spectrum and Other Conditions*. Lenexa, KS: AAPC Publishing.

[B46] Parker D. R. Boutelle K. (2009). Executive function coaching for college students with learning disabilities and ADHD: a new approach for fostering self-determination. *Learn. Disabil. Res. Pract.* 24 204–215. 10.1111/j.1540-5826.2009.00294.x

[B47] Parker D. R. Hoffman S. F. Sawilowsky S. Rolands L. (2011). An examination of the effects of ADHD coaching on university students’ executive functioning. *J. Postsecond. Educ. Disabil.* 24 115–132.

[B48] Pillay Y. Bhat C. S. (2012). Facilitating support for students with Asperger’s syndrome. *J. Coll. Stud. Psychother.* 26 140–154.

[B49] Prevatt F. Yelland S. (2015). An empirical evaluation of ADHD coaching in college students. *J. Atten. Disord.* 19 666–677. 10.1177/1087054713480036 23509112

[B50] Prince D. E. (2010). An exceptional path: an ethnographic narrative reflecting on autistic parenthood from evolutionary, cultural, and spiritual perspectives. *Ethos* 38 56–68. 10.1111/j.1548-1352.2009.01081.x

[B51] Pugliese C. E. White S. W. (2014). Brief report: problem solving therapy in college students with autism spectrum disorders: feasibility and preliminary efficacy. *J. Autism Dev. Disord.* 44 719–729. 10.1007/s10803-013-1914-8 23963592

[B52] Robbins A. (2011). *The Geeks Shall Inherit the Earth: Popularity, Quirk Theory, and Why Outsiders Thrive After High School*. London: Hachette UK.

[B53] Roberts E. L. Ju S. Zhang D. (2016). Review of practices that promote self-advocacy for students with disabilities. *J. Disabil. Policy Stud.* 26 209–220. 10.1177/1044207314540213

[B54] Roberts N. Birmingham E. (2017). Mentoring university students with ASD: a mentee-centered approach. *J. Autism Dev. Disord.* 47 1038–1050. 10.1007/s10803-016-2997-9 28132120

[B55] Rothman T. Maldonado J. M. Rothman H. (2008). Building self-confidence and future career success through a pre-college transition program for individuals with disabilities. *J. Vocat. Rehabil.* 28 73–83.

[B56] Schindler V. Cajiga A. Aaronson R. Salas L. (2015). The experience of transition to college for students diagnosed with Asperger’s disorder. *Open J. Occup. Ther.* 3 2 10.15453/2168-6408.1129

[B57] Shattuck P. T. Narendorf S. C. Cooper B. Sterzing P. R. Wagner M. Taylor J. L. (2012a). Postsecondary education and employment among youth with an autism spectrum disorder. *Pediatrics* 129 1042–1049. 10.1542/peds.2011-2864 22585766 PMC3362908

[B58] Shattuck P. T. Orsmond G. I. Wagner M. Cooper B. P. (2011a). Participation in social activities among adolescents with an autism spectrum disorder. *PLoS ONE* 6:e27176 10.1371/journal.pone.0027176 PMC3215697 22110612

[B59] Shattuck P. T. Roux A. M. Hudson L. E. Taylor J. L. Maenner M. J. Trani J. F. (2012b). Services for adults with an autism spectrum disorder. *Can. J. Psychiatry* 57 284–291. 22546060 10.1177/070674371205700503 PMC3538849

[B60] Shattuck P. T. Wagner M. Narendorf S. Sterzing P. Hensley M. (2011b). Post-high school service use among young adults with an autism spectrum disorder. *Arch. Pediatr. Adolesc. Med.* 165 141–146. 10.1001/archpediatrics.2010.279 21300654 PMC3097532

[B61] Shattuck P. T. Steinberg J. Yu J. Wei X. Cooper B. P. Newman L. (2014). Disability identification and self-efficacy among college students on the autism spectrum. *Autism Res. Treat.* 2014:924182 10.1155/2014/924182 PMC3953486 24707401

[B62] Shattuck P. T. Seltzer M. M. Greenberg J. S. Orsmond G. I. Bolt D. Kring S. (2007). Change in autism symptoms and maladaptive behaviors in adolescents and adults with an autism spectrum disorder. *J. Autism Dev. Disord.* 37 1735–1747. 10.1007/s10803-006-0307-7 17146700 PMC3265360

[B63] Shogren K. A. Plotner A. J. (2012). Transition planning for students with intellectual disability, autism, or other disabilities: data from the National Longitudinal Transition Study-2. *Intellect. Dev. Disabil.* 50 16–30. 10.1352/1934-9556-50.1.16 22316223

[B64] Shore S. M. (Ed.) (2004). *Ask and Tell: Self-Advocacy and Disclosure for People on the Autism Spectrum.* Lenexa, KS: AAPC Publishing.

[B65] Spain D. Blainey S. H. (2015). Group social skills interventions for adults with high-functioning autism spectrum disorders: a systematic review. *Autism* 19 874–886. 10.1177/1362361315587659 26045543

[B66] Spielberger C. D. Gorsuch R. L. Lushene R. E. Vagg R. R. Jacobs G. A. (1983). *Manual for the State-Trait Anxiety Inventory (Form Y)*. Palo Alto, CA: Consulting Psychologists Press.

[B67] Test D. W. Fowler C. H. Brewer D. M. Wood W. M. (2005a). A content and methodological review of self-advocacy intervention studies. *Except. Child.* 72 101–125. 10.1177/001440290507200106

[B68] Test D. W. Fowler C. H. Wood W. M. Brewer D. M. Eddy S. (2005b). A conceptual framework of self-advocacy for students with disabilities. *Remedial Spec. Educ.* 26 43–54. 10.1177/07419325050260010601

[B69] Thompson B. (1998). Statistical significance and effect size reporting: portrait of a possible future. *Res. Sch.* 5 33–38.

[B70] US Census Bureau (2012). *Statistical Abstract of the United States: 2012*, 13 1st Edn Washington, DC: US Census Bureau.

[B71] Van Bergeijk E. Klin A. Volkmar F. (2008). Supporting more able students on the autism spectrum: College and beyond. *J. Autism Dev. Disord.* 38 1359–1370. 10.1007/s10803-007-0524-8 18172747

[B72] Van Hees V. Moyson T. Roeyers H. (2015). Higher education experiences of students with autism spectrum disorder: challenges, benefits and support needs. *J. Autism Dev. Disord.* 45 1673–1688. 10.1007/s10803-014-2324-2 25448918

[B73] Wehman P. Schall C. Carr S. Targett P. West M. Cifu G. (2014). Transition from school to adulthood for youth with autism spectrum disorder: what we know and what we need to know. *J. Disabil. Policy Stud.* 25 30–40. 10.1177/1044207313518071

[B74] Wei X. Jennifer W. Y. Shattuck P. McCracken M. Blackorby J. (2013). Science, technology, engineering, and mathematics (STEM) participation among college students with an autism spectrum disorder. *J. Autism Dev. Disord.* 43 1539–1546. 10.1007/s10803-012-1700-z 23114569 PMC3620841

[B75] Wigham S. McConachie H. Tandos J. Le Couteur A. S. Gateshead Millennium Study Core Team (2012). The reliability and validity of the social responsiveness scale in a UK general child population. *Res. Dev. Disabil.* 33 944–950. 10.1016/j.ridd.2011.12.017 22277583

[B76] Wenzel C. Rowley L. (2010). Teaching social skills and academic strategies to college students with Asperger’s syndrome. *Teach. Except. Chil.* 42 44–51. 10.1177/004005991004200505

[B77] White G. W. Summers J. A. Zhang E. Renault V. (2014). Evaluating the effects of a self-advocacy training program for undergraduates with disabilities. *J. Postsecond. Educ. Disabil.* 27 229–244.

[B78] White S. W. Ollendick T. H. Bray B. C. (2011). College students on the autism spectrum: prevalence and associated problems. *Autism* 15 683–701. 10.1177/1362361310393363 21610191

[B79] Yoo H. J. Bahn G. Cho I. H. Kim E. K. Kim J. H. Min J. W. (2014). A randomized controlled trial of the Korean version of the PEERS^®^ parent-assisted social skills training program for teens with ASD. *Autism Res.* 7 145–161. 10.1002/aur.1354 24408892

[B80] Zeedyk S. M. Tipton L. A. Blacher J. (2014). Educational supports for high functioning youth with ASD: The postsecondary pathway to college. *Focus Autism Other Dev. Disabil.* 31 37–48. 10.1177/1088357614525435

[B81] Zimet G. D. Dahlem N. W. Zimet S. G. Farley G. K. (1988). The multidimensional scale of perceived social support. *J. Pers. Assess.* 52 30–41. 10.1207/s15327752jpa5201_2 2280326

